# Differential effects of synthetic media on long-term growth, starch accumulation and transcription of ADP-glucosepyrophosphorylase subunit genes in *Landoltia punctata*

**DOI:** 10.1038/s41598-019-51677-w

**Published:** 2019-10-25

**Authors:** Chokchai Kittiwongwattana

**Affiliations:** Department of Biology, Faculty of Science, King Mongkut’s Institute of Technology Ladkrabang, Bangkok, 10520 Thailand

**Keywords:** Plant molecular biology, Plant physiology

## Abstract

Murashige & Skoog (MS) and Hoagland’s media were previously used for *in vitro* culture of *Landoltia punctata*. During subsequent *ex vitro* culture, the use of MS medium resulted in a higher growth rate, compared to Hoagland’s medium. Thus, a higher starch content of *L. punctata* in MS medium was previously hypothesized. Here, *L. punctata* strain 5632 was isolated and characterized using morphological characteristics and the *atpF*-*atpH* intergenic region. During early cultivation stage, fresh weight and relative growth rate in MS medium were lower than Hoagland’s medium. Conversely, starch content in MS medium was considerably higher than in Hoagland’s medium. Medium effects on expression of genes coding for starch-biosynthesis ADP-glucosepyrophosphorylase (AGPase) were determined. Genomic fragments of small (*LeAPS*) and large (*LeAPL1*) AGPase subunits were characterized. Differential expression between each AGPase subunit genes was observed in both media. Additionally, in MS medium, the highest correlation coefficients between starch content and gene expression was found with *LeAPS* (0.81) and followed by *LeAPL3* (0.67), *LeAPL2* (0.65) and *LeAPL1* (0.28). In Hoagland’s medium, the coefficients of *LeAPL3* (0.83) and *LeAPL2* (0.62) were higher than *LeAPS* (0.18) and *LeAPL1* (−0.62). This suggested different levels of contributions of these genes in starch biosynthesis in both media.

## Introduction

Starch functions as an important energy reserve in plants^1^. During photosynthesis, carbon compounds are generated and converted into glucose that serves as the precursor for starch formation^1^. There are three committed steps in the process^1^. The first one is the generation of ADP-glucose. Secondly, the glucosyl moiety of ADP-glucose is linked to an existing glucan chain through the formation of the α(1-4) linkage, resulting in an extension of the starch chain. Finally, branching of the chain is formed through the formation of the α(1-6) linkage between glucosyl moieties and the chain. ADP-glucose pyrophophorylase (AGPase) is responsible for the formation of ADP-glucose that was proposed as the rate-limiting step^2^. In higher plants, AGPase is heterotetrameric and consists of two small and two large subunits^3,4^. Small subunits play the catalytic role, while large subunits mainly function in regulating the enzyme activity^5,6^. Genes that code for large and small subunits were previously identified in various dicotyledonous and monocotyledonous plants, e.g., *Arabidopsis*^7^, potato^8,9^, chickpea^10^, maize^11,12^ and wheat^13,14^. Additionally, overexpression of AGPase large subunit genes increased starch content in maize and wheat grains^12,15^. Another study showed that a transgenic wheat line, overexpressing an AGPase small subunit gene, accumulated starch at a level substantially higher than the wild-type cultivar^6^.

Duckweeds are small aquatic plants that belong to the family Lemnaceae^16^. Thus far, 37 duckweed species were affiliated with the family and classified into five genera, including *Spirodela*, *Landoltia*, *Lemna*, *Wolffiella* and *Wolffia*^16,17^. Fronds are leaf-like and function as both vegetative and reproductive organs^18^. *L. punctata* has gained the interest in research communities, because of its high starch content that could be used in bioethanol production^19–21^. Several advantages of *L. punctata* over other energy crops were also cited^22^. These included rapid growth rate, high starch content and low levels of fiber and lignin content^22^. Additionally, the use of *L. punctata* for the energy industry did not compete for land with food crops^23^. Various external and internal factors affected starch biosynthesis of *L. punctata*. Nutrient starvation induced starch accumulation^4^, while phytohormones were internal regulators of starch biosynthesis^24,25^. Expression of *L. punctata* AGPase subunit genes, including *LeAPS*, *LeAPL1*, *LeAPL2* and *LeAPL3*, were also affected by growth conditions^4,25^. Expression of *LeAPL2* under nitrogen starvation was relatively lower than that under phosphorus starvation^4^. In contrast, nitrogen deficiency induced an early response of *LeAPL3*, while phosphorus deprivation resulted in an upregulation of *LeAPL3* at a later phase^4^. A transcriptomic study showed that, when *L. punctata* was treated with uniconazole, expression of two AGPase large subunit genes and starch content were concurrently increased^25^.

Synthetic media were generally used for culturing duckweeds under controlled environments^26,27^. Medium compositions had various physiological impacts on duckweeds. In *Spirodela polyrhiza*, exogenous addition of abscisic acid (ABA) in culture medium induced a morphological transition of fronds to turions, a starch accumulating structure^28^. *SpAPL2* and *SpAPL3*, encoding two different AGPase large subunits, were also upregulated under such conditions, whereas *SpAPL1* expression was not significantly increased^28^. For *L. punctata*, biomass production was higher when plants were cultivated in Hoagland’s medium for 13 days, compared to MS medium^26^. However, after transferred to nutrient-limited pond water for *ex vitro* culture, *L. punctata* obtained from MS medium grew 17.1% faster than those from Hoagland’s medium. It was previously hypothesized that cultivation in MS medium resulted in higher starch accumulation that subsequently promoted *ex vitro* growth^26^. This hypothesis was tested here. *L. punctata* strain 5632 was newly isolated and characterized, based on its morphologies and the *atpF*-*atpH* intergenic region sequence. Strain 5632 was long-term (35 days) cultured in MS and Hoagland’s media to observe its biomass production and starch accumulation. The cDNA fragments of *LeAPS* and *LeAPL1*, coding for AGPase large and small subunits, respectively, were cloned and compared with the reference sequences of *L. punctata* strain 0202^4^. Genomic fragments of both genes were also cloned, and their introns and exons were characterized. Expression levels of four AGPase subunit genes, including *LeAPS*, *LeAPL1*, *LeAPL2* and *LeAPL3*, in both media were analysed. Correlation coefficients, between AGPase gene expression levels and starch content, were calculated to determine the contribution of each gene in starch biosynthesis, during the cultivation in MS and Hoagland’s media.

## Results and Discussion

### Morphological and molecular characterization

**Figure 1:**
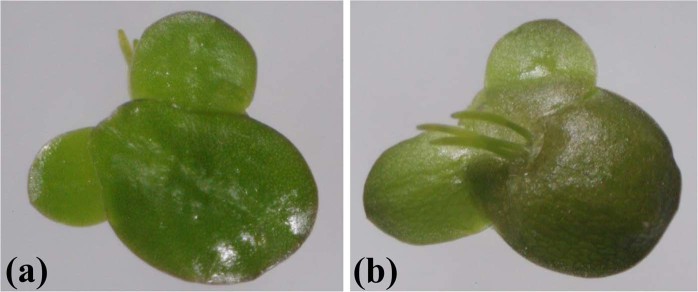
The upper (**a**) and lower (**b**) sides of strain 5632.

**Figure 2:**
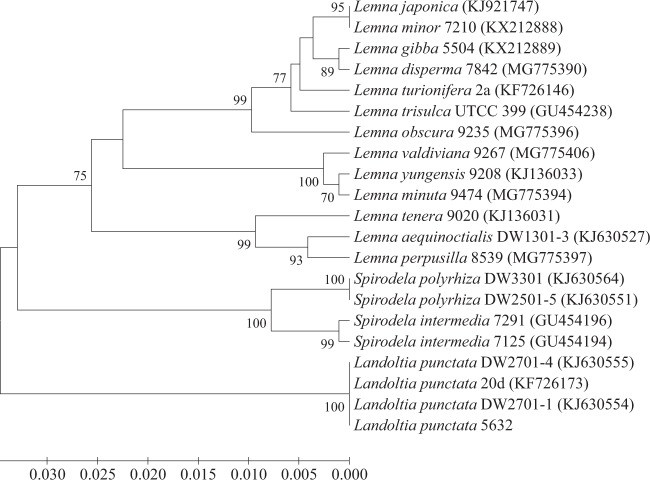
Phylogenetic relationship between strain 5632 and selected members in the family Lemnaceae. Reconstruction of the phylogenetic tree was based on the sequence of the *atpF-atpH* intergenic region, using the UPGMA method. Bootstrap values are shown at tree nodes as the percentage of 1,000 replicates. Only values equal to or higher than 70% are shown. Bar indicated the evolutionary distance as the number of base substitutions per site.

Duckweed samples were collected, and an axenic culture on Hoagland’s medium was established from a single mother frond to ensure the genetic similarity among samples throughout the study. The strain was designated as strain 5632. It was important to note here that the nutrient compositions of Hoagland’s medium were somewhat different from the original publication^29^. Originally, two different macronutrient solutions were described for the preparation of Hoagland’s medium^29^. The main difference between them was the nitrogen source. While one solution contained only NO_3_^−^, the other one was supplemented with both NO_3_^−^ and NH_4_^+^ ^29^. Hoagland’s solution, used in this study, had nutrient compositions (Table 1) that were more similar to the latter one. However, there was a distinction in the NO_3_^−^ concentration in the original solution (1 mM)^29^ and this study (6 mM). Additionally, iron was supplied as iron tartate in the original publication^29^, whereas it was given here as chelated iron. Basic morphological characteristics of strain 5632 were examined (Fig. 1). Fronds were inflated and oval-shaped. The upper surface was dark green. The lower surface was reddish, suggesting the accumulation of anthocyanins in the tissues. Several roots were present on the lower side of the fronds, indicating that strain 5632 was likely a member of the genus *Landoltia*. The presence and the number of roots were distinguishing morphological characteristics among the five genera of the family Lemnaceae^30^. For example, a single root was present on each frond of *Lemna* spp., whereas plants in genera *Wolffia* and *Wolffiella* did not produce roots. In contrast, several roots were present on each frond of members in genera *Spirodela* and *Landoltia*^31^. The number of roots of the genus *Spirodela* ranged from 7 to 21, while that of the genus *Landoltia* ranged from 2 to 7^32^. Additional characteristics, e.g., a medial series of papillae on the upper surface, frond prophyllum, frond nerves and external anther locules, could be used to differentiate *Landoltia* from *Spirodela*^32^. However, because of their small and highly reduced structures, identification of duckweeds at the species levels could be challenging without a special expertise in the Lemnaceae family^33^. Biochemical and DNA sequence data were previously combined with morphological and anatomical markers to study the phylogeny of the family Lemnaceae^34^. The phylogenetic tree confirmed the presence of the paraphyletic subfamily *Lemnoideae*, consisting of *Spirodela*, *Landoltia*, and *Lemna*, and the monophyletic subfamily *Wolffioideae*, comprising of *Wolffia* and *Wolffiella*. Additionally, a DNA barcode, based on the *atpF*-*atpH* intergenic region, was developed to aid the characterization of lemnaceous plants^33^. For identification of strain 5632, the *atpF*-*atpH* intergenic region was amplified from plastid DNA and sequenced. Using the blastn analysis, the highest identity level (100%) was found between the sequences of strain 5632 and *L. punctata* strains DW2701-1 (accession number: KJ630554) and DW2701-4 (KJ630555). To understand the phylogenetic relationship between strain 5632 and other *L. punctata* strains, the phylogenetic tree was reconstructed based on the *atpF*-*atpH* intergenic sequences. It showed that strain 5632 was closely related to *L. punctata* strains DW2701-1, DW2701-4 and 20d (Fig. 2). This was supported by the 100% confidence level. Consistent with its morphologies, the molecular characterization indicated that strain 5632 was a member of the species *L. punctata*.

**Table 1:** Compositions of MS and Hoagland’s media.

Nutrients	Concentrations (mM)	
	MS medium	Hoagland’s medium
KNO_3_	18.8	6
NH_4_H_2_PO_4_	—	1
MgSO_4_	1.5	2
KH_2_PO_4_	1.2	—
NH_4_NO_3_	20.6	—
CaCl_2_	3	—
Ca(NO_3_)_2_	—	4
H_3_BO_3_	0.1	4.6 × 10^−2^
Na_2_MoO_4_·2H_2_O	1 × 10^−3^	—
MnSO_4_·H_2_O	0.1	—
ZnSO_4_·7H_2_O	3 × 10^−2^	7.7 × 10^−4^
CuSO_4_·5H_2_O	1 × 10^−4^	3.2 × 10^−4^
CoCl_2_·6H_2_O	1 × 10^−4^	—
FeSO_4_·7H_2_O	0.1	9 × 10^−3^
MnCl_2_·4H_2_O	—	9.2 × 10^−3^
MoO_3_	—	1.1 × 10^−4^
KI	5 × 10^−3^	—
Na_2_EDTA·2H_2_O	0.1	9 × 10^−3^
myo-Inositol	0.6	—
Nicotinic acid	4.1 × 10^−3^	—
Pyridoxine-HCl	2.4 × 10^−3^	—
Glycine	2.7 × 10^−2^	—
Thiamine·HCl	3 × 10^−4^	—

### Growth and starch accumulation of *L. punctata* 5632 in synthetic media

**Figure 3:**
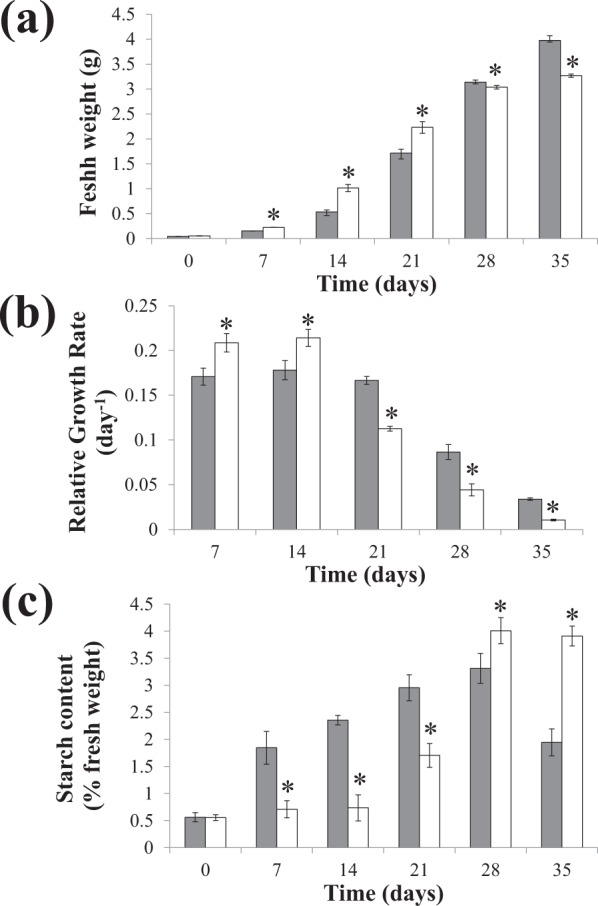
Fresh weight (**a**), relative growth rate (**b**) and starch content (**c**) of *L. punctata* strain 5632 grown in MS (gray bars) and Hoagland’s (white bars) media. Standard deviation (*n* = 3) is shown by vertical bars. Asterisks indicate statistically significant differences (*P* < 0.05) between the parameters in MS and Hoagland’s media on each day.

A previous study showed that the growth rate of *L. punctata* in MS medium was lower than that in Hoagland’s medium^26^. However, after transferred to nutrient-limited water, *L. punctata* obtained from MS medium grew at a higher rate than those from Hoagland’s medium^26^. This suggested a higher starch content of *L. punctata* in MS medium that could be used to sustain subsequent *ex vitro* growth. To test this hypothesis, strain 5632 was grown in MS and Hoagland’s liquid media for 35 days, and fresh weight was determined every seven days (Fig. 3a). It was important to note that there were some differences, e.g. types of some micronutrient salts and some nutrient concentrations, between the compositions of Hoagland’s solution used here (Table 1) and in the previous study^26^. However, during the first 14 days of cultivation, the fresh weight of strain 5632 in Hoagland’s solution was higher than in MS medium. This was consistent with the previous study^26^. This continued until day 21 of cultivation. Conversely, on day 28, the fresh weight in MS medium (3.14 ± 0.04 g) became significantly higher (*P* = 0.038) than that in Hoagland’s medium (3.04 ± 0.04 g). On day 35, it became 1.2 times of that in Hoagland’s medium.

Relative growth rate of strain 5632 in both media were calculated and found relatively consistent with fresh weight analysis (Fig. 3b). During the first 14 days of cultivation, strain 5632 in MS medium exhibited lower relative growth rate, compared to Hoagland’s medium. In contrast, on day 28 and 35 of cultivation, the relative growth rate in MS medium became substantially higher than in Hoagland’s medium. This indicated a more rapid reduction in growth of strain 5632 in Hoagland’s medium. A discrepancy between fresh weight and relative growth rate was observed on day 21. While the fresh weight in MS medium was lower than Hoagland’s medium (Fig. 3a), the relative growth rate became considerably higher than Hoagland’s medium (Fig. 3b). This was because of the differential increase between the fresh weight in both media. From day 14 to day 21, the fresh weight in MS medium elevated from 0.53 ± 0.04 g to 1.71 ± 0.08 g, respectively. This yielded the relative growth rate at 0.17 ± 0.004 day^−1^. During the same period, the fresh weight in Hoagland’s medium rose from 1.02 ± 0.07 g to 2.23 ± 0.12 g, and yielded relative growth rate at 0.11 ± 0.002 day^−1^.

Starch content of strain 5632 in MS and Hoagland’s media was also determined throughout the cultivation period (Fig. 3c). In MS medium, it continuously increased and reached the level of 3.31 ± 0.28% on day 28. This was followed by a decrease to 1.95 ± 0.25% on day 35. In contrast, starch content of strain 5632 in Hoagland’s medium increased relatively slowly during the first three weeks of cultivation. Subsequently, it reached the highest level (4.01 ± 0.24%) on day 28 and slightly decreased to 3.91 ± 0.18% on day 35. Comparison between the fresh weight and starch content in both media revealed an inverse correlation. From day 7 to 21, the fresh weight of strain 5632 in MS medium was lower than Hoagland’s medium, whereas starch content in MS medium was higher than the other medium. The opposite was found on day 28 and 35 of cultivation.

Growth and starch content of strain 5632 were likely affected by the differences between the concentrations of both NH_4_^+^ and NO_3_^−^ in MS and Hoagland’s media (Table 1). During the early phase of cultivation, the slower growth in MS medium was likely caused by the high concentration of NH_4_^+^ in MS medium (20.6 mM). A previous study showed that NH_4_^+^ was preferable over NO_3_^−^ for the uptake by *L. punctata*^35^. Thus, cultivation of strain 5632 in the medium likely caused excessive accumulation of NH_4_^+^ in strain 5632. This resulted in lower biomass production, compared to the use of Hoagland’s medium, that was supplemented with 1 mM NH_4_^+^. When present at a high concentration, NH_4_^+^ was known to negatively affect plant growth^36^. This was also demonstrated in other duckweed species. Relative growth rate of *S. polyrhiza* decreased upon the increase of NH_4_^+^ concentration^37^. Growth inhibition and frond chlorosis were also found in *Lemna minor*, grown in Hoagland’s medium supplemented with 280 and 840 mg l^−1^ NH_4_^+^-N, which equaled to 20 and 60 mM NH_4_^+^, respectively^38^. During the later cultivation period, growth of strain 5632 in Hoagland’s medium declined more rapidly, compared to that in MS medium. This may be attributed to the NO_3_^−^ concentration in Hoagland’s medium (10 mM) that was only 25.4% of that in MS medium (39.4 mM). The result suggested that this level of NO_3_^−^, in Hoagland’s medium, was insufficient to sustain growth of strain 5632 for the entire cultivation period. Additionally, another nutrient that may cause the distinction in growth of strain 5632 in the two media was iron. It is an important microelement in various plant metabolic processes^39^. Generally, FeSO_4_ was added together with Na_2_EDTA to generate chelated iron that ensured its transport into plant cells^40^. Here, the concentrations of FeSO_4_ and Na_2_EDTA, in Hoagland’s medium, were approximately 9% of those in MS medium and may be insufficient to sustain growth during the extended cultivation period. On the other hand, starch biosynthesis was previously known to be upregulated under nutrient starvation conditions, which also resulted in growth retardation^4,5,22,41,42^. This was somewhat consistent with the increase in starch content of strain 5632 in MS medium, where growth was relatively lower than in Hoagland’s medium. However, as mentioned above, both NH_4_^+^ and NO_3_^−^ in MS medium were supplied at much higher levels than Hoagland’s medium. This suggested that differential starch accumulation of strain 5632 in the two media was likely a secondary effect that resulted from growth modulation by the nitrogen sources.

### Nucleotide variations in full-length cDNA sequences of *LeAPL1* and *LeAPS*

**Figure 4:**
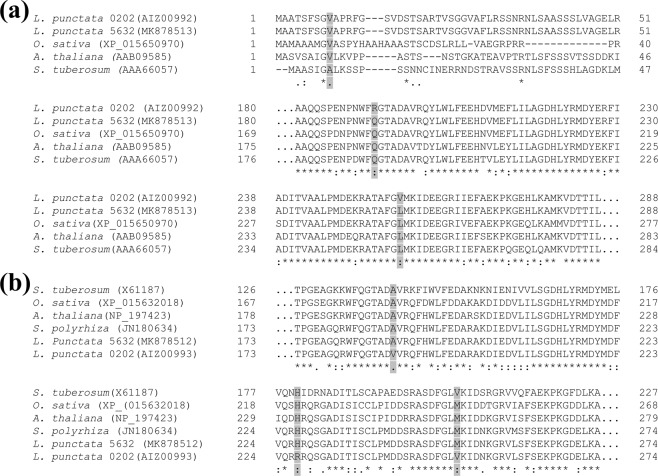
Multiple alignment of deduced amino acid sequences of of LeAPS (**a**) and LeAPL1 (**b**) of *L. punctata* strain 5632, *L. punctata* strain 0202 and their orthologs in other plant species. Amino acid variations between *L. punctata* strains 5632 and 0202 are highlighted.

Full-length *LeAPS* and *LeAPL1* cDNA were previously sequenced in *L. punctata* strain 0202 that was collected in China^4^. However, strain 5632 was obtained from a local pond in Thailand. Thus, cDNA sequences of both genes were determined to investigate nucleotide variations that may exist between the two genes of these strains. Full-length *LeAPS* (1,578 bp) and *LeAPL1* (1,554 bp) cDNA of strain 5632 were sequenced and compared with their corresponding sequences of strain 0202 (*LeAPS*: KJ603243; *LeAPL1*: KJ603244)^4^. For *LeAPS*, three nucleotide variations were found at positions 27, 575 and 769 (Table 2). Two of these resulted in changes of the deduced amino acid residues. For *LeAPL1*, there were three nucleotide variations at positions 563, 680 and 754, all of which resulted in changes of corresponding amino acid residues (Table 2). To investigate whether these amino acid variations were inherent to strain 5632, the deduced LeAPL1 and LeAPS protein sequences of strain 5632 were used for multiple alignment analysis with those of strain 0202 and their orthologs from other plant species. The analysis indicated that amino acid positions 9, 192 and 257 of LeAPS (Fig. 4a) and 188 and 227 of LeAPL1 (Fig. 4b) of strain 5632 were more similar to the orthologs than strain 0202. These differences between the deduced amino acid sequences of strains 5632 and 0202 were possibly derived from DNA amplification errors during the cloning step. *LeAPS* and *LeAPL1* cDNA of strain 0202 were amplified, using rTaq DNA polymerase^4^. In contrast, the cloned sequences of strain 5632 were obtained with Q5 high-fidelity DNA polymerase, of which the error rate was described as 280 times lower than regular *Taq* DNA polymerase (www.neb.com). Thus, these amino acids of strain 5632 likely represented the actual residues of LeAPS and LeAPL1. The only exception was the amino acid position 252 of LeAPL1 that was derived from its corresponding codon containing the nucleotide variant position 754. The amino acid sequences of strain 5632 and other orthologs carried methionine at this position, as opposed to valine found in *L. punctata* 0202 and *S. tuberosum* sequences (Fig. 4b). Thus, this nucleotide position may represent a genotypic variation between *LeAPL1* of strains 5632 and 0202.

**Table 2:** Nucleotide variants found in *LeAPS* and *LeAPL1* cDNA of *L. punctata* 5632, comparing to the reference sequences of *L. punctata* 0202. The variants are underlined and shown with their associated codons. Corresponding amino acid residues are also indicated.

Gene	Nucleotide positions	Amino acid positions	*L. punctata* 5632		*L. punctata* 0202	
			Codons	Amino acid	Codons	Amino acid
*LeAPS*	27	9	GTT	(valine)	GTC	(valine)
	575	192	CAG	(glutamine)	CGG	(arginine)
	769	257	CTG	(leucine)	GTG	(valine)
*LeAPL1*	563	188	GCA	(alanine)	GTA	(valine)
	680	227	CAT	(histidine)	CGT	(arginine)
	754	252	ATG	(methionine)	GTG	(valine)

### Organization of genomic *LeAPS* and *LeAPL1*

**Figure 5:**
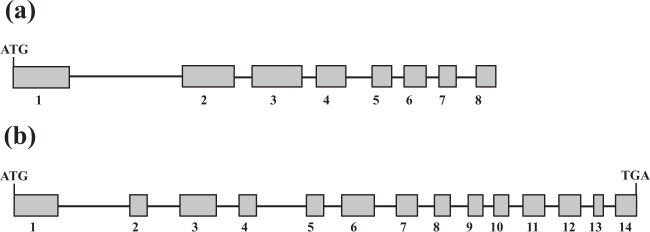
Structural organizations of genomic *LeAPS* (**a**) and *LeAPL1* (**b**). Exons (gray boxes) and introns (black lines) are shown. Exon numbers are indicated below the diagrams. ATG represents the start codon, while TGA is the stop codon.

Although their cDNA sequences were previously characterized^4^, the genomic sequences of *LeAPS* and *LeAPL1* were still unavailable. Here, amplification of genomic *LeAPS* with the CLeAPS-F and LeAPS-R primers was successful. The length of the amplified fragment was 2,621 bp. It was important to note that the fragment was only partial and did not cover the remaining 128 nucleotides on the 3′ region of its cDNA. Eight exons and seven introns were identified in the sequence (Fig. 5a). The numbers of exons and introns found in genes coding for AGPase small subunits were variable among different plant species. A study on the genomic structure of maize *Bt2* showed that it consisted of ten exons and nine introns^11^. In contrast, *sAGP* of potato^43^ and *ibAGP1* and *ibAGP2* of sweet potato^44^ were similarly organized into nine exons and eight introns. On the other hand, the genomic fragment of *LeAPL1* (3,161 bp) was obtained using the CLeAPL1-F and CLeAPL1-R primers. The genomic sequence consisted of 14 exons and 13 introns (Fig. 5b). The average length of the exons was 111 bp. Exon 1 was the longest (210 bp), while exon 13 was the shortest (61 bp). The organization of exons and introns in *LeAPL1* was different from its ortholog, *SpAPL1* of *S. polyrhiza*, where 15 exons and 14 introns were characterized^28^.

### Expression of AGPase subunit genes and correlation with starch accumulation

**Figure 6:**
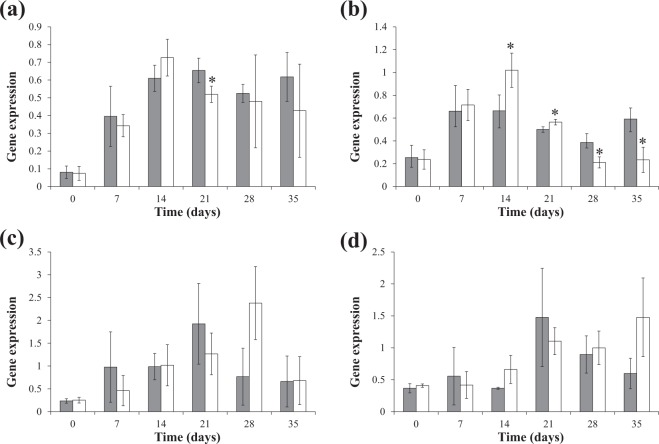
Expression levels of *LeAPS* (**a**), *LeAPL1* (**b**), *LeAPL2* (**c**) and *LeAPL3* (**d**) of *L. punctata* strain 5632, grown in MS (gray bars) and Hoagland’s (white bars) media. Standard deviation (*n* = 3) is shown by vertical bars. Asterisks indicate statistically significant differences (*P* < 0.05) between expression levels of each AGPase subunit genes in MS and Hoagland’s media on each day.

Expression of AGPase subunit genes, including *LeAPS*, *LeAPL1*, *LeAPL2* and *LeAPL3*, was analysed, in order to understand the molecular responses towards nutrient compositions in MS and Hoagland’s media. Based on their genomic sequences, new primers were designed and used to quantify expression of *LeAPS* and *LeAPL1*. Unlike the previously described primers^4^, they spanned over intron regions to ensure the amplified products were derived from cDNA of processed mRNA molecules. For *LeAPL2* and *LeAPL3*, the primers used in the analysis were from the previous study^4^, because their genomic sequences were unavailable. Additionally, the expression analysis here covered the cultivation period of five weeks. This was much longer than other previous studies where it ranged from seven to ten days^4,25,42^ and enabled the monitoring of the long-term gene regulation in both media. Expression levels of each gene during the cultivation in both media are shown in Fig. 6. In MS medium, the highest expression levels of *LeAPS* (0.65 ± 0.07), *LeAPL2* (1.92 ± 0.88) and *LeAPL3* (1.48 ± 0.77) were observed on day 21 and approximately 8.2 times of the level on day 0. In contrast, *LeAPL1* expression increased and reached the maximum level (0.66 ± 0.14) on day 14, which was approximately 2.6 times of that on day 0. Similar to *LeAPS*, the highest levels of *LeAPL2* and *LeAPL3* expression occurred on day 21 and were 4.0 and 8.1 folds of day 0, respectively. In Hoagland’s medium, expression of *LeAPS* (0.72 ± 0.10) and *LeAPL1* (1.02 ± 0.15) similarly peaked on day 14 and were 10.3 and 4.2 folds of day 0, respectively. *LeAPL2* expression became the highest on day 28 (2.38 ± 0.80) and was 9.5 times of day 0. In contrast, expression of *LeAPL3* continuously increased throughout the cultivation period and was the highest on day 35 (1.48 ± 0.61) and 3.6 folds of day 0. Significant differences were also observed between expression levels of some AGPase subunit genes in both media. On day 21, *LeAPS* in MS medium was expressed at a level significantly higher (*P* = 0.049) than in Hoagland’s medium. On the other hand, *LeAPL1* in MS medium was expressed at levels significantly lower than in Hoagland’s medium on day 14 (*P* = 0.039) and 21 (*P* = 0.029). However, its expression in MS medium became significantly higher than in Hoagland’s medium on day 28 (*P* = 0.03) and 35 (*P* = 0.014). In contrast, differences in *LeAPL2* and *LeAPL3* expression levels observed in MS and Hoagland’s media were not substantial throughout the cultivation period. The result observed here indicated differential regulation of each AGPase subunit gene. This was also previously found with *L. punctata* strain 0202, that was grown under nitrogen and phosphorus starvation conditions^4^. In response to nitrogen starvation, upregulation of *LeAPL1* and *LeAPL3* was observed earlier than *LeAPL2*^4^. Under phosphorus deprivation, a dramatic increase in *LeAPL1* and *LeAPL2* expression occurred more rapidly than *LeAPL2*^4^. In contrast, *LeAPS* was highly expressed throughout the nitrogen starvation period and during the early stage of phosphorus starvation^4^. ABA may play a role in regulating expression of AGPase genes in this study, since its application on *L. punctata* was previously shown to increase starch content and AGPase activity^20,45^. Consistently, a transcriptomic study demonstrated that both ABA content and expression levels of AGPase large subunit genes were elevated, when *L. punctata* was treated with uniconazole^24,25^. Reports in *S. polyrhiza*^28^ and rice cell culture^46^ suggested that each AGPase subunit gene may respond to ABA differently. Expression of *SpAPL2* and *SpAPL3* was upregulated by the ABA treatment^28^. In contrast, the expression level of *SpAPL1* remained relatively unchanged and was much lower than *SpAPL2* and *SpAPL3*. On the other hand, application of ABA to rice cell culture resulted in elevated expression of *OsAPL3* and *OsAPS1*, while its effects on other rice AGPase subunit genes were much less pronounced^46^. These previous observations suggested that ABA effects on expression of AGPase genes may also vary among different plant species.

Because of their differential expression, each AGPase subunit genes may contribute to starch accumulation of strain 5632 in MS and Hoagland’s media differently. Correlation coefficients between the expression levels of AGPase subunit genes and the starch content were determined. In MS medium, *LeAPS* had the highest correlation coefficient (0.81) and was followed by *LeAPL3* (0.67) and *LeAPL2* (0.65). A small correlation (0.28) was observed with *LeAPL1*. In Hoagland’s medium, expression of *LeAPL3* was the most correlated (0.83) with starch content. An intermediate level of correlation was found with *LeAPL2* (0.62). In contrast, the correlation coefficient of *LeAPS* was only 0.18, while *LeAPL1* was negatively correlated (−0.62). The intermediate and high correlation coefficients of *LeAPL2* and *LeAPL3* were consistent with their expression levels that were found comparable in both media (Fig. 6c,d). This suggested the roles of *LeAPL2* and *LeAPL3* in starch biosynthesis may be non-specific to the media. In contrast, *LeAPS* contribution may be more pronounced in MS medium. On the other hand, the correlation coefficients of *LeAPL1* in the two media were highly distinctive and ranged from low to negatively intermediate levels. This indicated its input in starch content was likely dependent on medium compositions. This was also reflected on the differences between its expression levels in both media (Fig. 6b). The correlation coefficients observed here were consistent with a previous study, where *L. punctata* strain 0202 was grown in Hoagland’s medium under nitrogen- and phosphorus-deficient conditions for seven days^4^. The correlations between *LeAPL2* and *LeAPL3* expression and starch content were either intermediate or high under both conditions. *LeAPS* was found correlated to starch accumulation specifically under phosphorus starvation. In contrast, low or negative correlations were also found with *LeAPL1* under nitrogen and phosphorus deprivation.

## Conclusions

Long-term (35 days) physiological and molecular responses were demonstrated in *L. punctata* strain 5632 cultured in MS and Hoagland’s media. During the early stage, the use of MS medium resulted in lower growth and higher starch content, compared to Hoagland’s medium. The situation was reverse during the last 14 days of the experiment. Expression of AGPase subunit genes was differentially regulated in both media. Based on the correlation coefficients between starch content and gene expression, *LeAPL2* and *LeAPL3* were likely important for starch biosynthesis in both media, while *LeAPS* was more specific to MS medium. In contrast, low or negative correlation coefficients were associated with *LeAPL1*.

## Methods

### Plant materials and cultivation

*L. punctata* (G. Mey.) Les & D. J. Crawford was collected from a local pond in Bangkok, Thailand. To establish *in vitro* culture, fronds were surface-sterilized, using 10% (v/v) NaClO solution and a few drops of Tween-20 for two minutes with vigorous shaking. They were washed in sterilized distilled water three times. Surface-sterilized fronds were grown on Hoagland’s solid medium (PhytoTechnology Laboratories, USA), containing 2% (w/v) sucrose and 0.7% (w/v) agar, pH 5.7. Growth conditions were 25 ± 2 °C and 16-hour-light/8-hour-dark photoperiods. Photosynthetically active radiation (PAR) flux was 65 µmol m^−2^ s^−1^. Daughter fronds derived from a single mother frond were obtained. Morphological characteristics were examined. This duckweed strain was registered to the Rutgers Duckweed Stock Cooperative as strain 5632.

### Determination of growth and starch content

Twenty colonies of strain 5632 were randomly selected and transferred to glass bottles containing 100 ml of either MS or Hoagland’s liquid media (Phytotechnology, USA), supplemented with 2% sucrose, pH 5.7. The cultures were grown for 35 days under 25 ± 2 °C and 16-hour-light/8-hour-dark photoperiods. PAR flux was 65 µmol m^−2^ s^−1^. Samples were collected on day 0, 7, 14, 21, 28 and 35 of cultivation and rinsed thoroughly with distilled water. Excessive water was removed by blotting on tissue paper. Total fresh weight was determined. Relative growth rate on day 7, 14, 21, 28 and 35 was calculated as follows:

$${\rm{Relative}}\,{\rm{growth}}\,{\rm{rate}}\,({{\rm{day}}}^{-1})=(\mathrm{ln}\,{W}_{t}\mbox{--}\,\mathrm{ln}\,{W}_{t-1})/{\rm{time}}$$

where *W*_*t*_ = fresh weight on day 7, 14, 21, 28 and 35 of cultivation; *W*_*t-1*_ = fresh weight of prior sample collection and time = the time interval (7 days).

One-hundred milligrams of fresh samples collected on each day were used for the analysis of starch content with Total Starch Assay Kit (Megazyme, Ireland), according to the manufacturer’s protocol. Water was used as the blank control, and 1 mg ml^−1^ D-glucose was used as the standard. The experiment was done in triplicate. Two-tailed student’s *t*-test was used to determine statistically significant differences (*P* < 0.05) between fresh weight, relative growth rate, and starch content, in MS and Hoagland’s media on each day.

### Amplification and phylogenetic analysis of the *atpF-atpH* intergenic region

Fronds from axenic culture were used for DNA extraction with a FavorPrep Plant Genomic DNA Extraction Mini Kit (Favorgen, Taiwan), according to the manufacturer’s protocol. Amplification and sequencing of the *atpF*-*atpH* intergenic region were done with the atpF-atpH forward (5′-ACTCGCACACACTCCCTTTCC-3′) and reverse (5′-GCTTTTATGGAAGCTTTAACAAT-3′) primers, according to the previous study^33^. The sequence was analysed with blastn. Multiple alignment of the sequence with those of other duckweed strains in the GenBank database used the CLUSTAL W program version 1.81^47^. Gaps were manually removed and adjusted. A phylogenetic tree was reconstructed, using unweighted pair group method with arithmetic mean (UPGMA), in MEGA 7.0^48^. Bootstrap analysis^49^ with 1,000 re-samplings determined the confidence level of each clade.

### Cloning and sequencing of *LeAPS* and *LeAPL1* cDNA

Total RNA was extracted from 100 mg of fronds, using a FavorPrep Plant Total RNA Mini Kit (Favorgen, Taiwan), according to the manufacturer’s protocol. Genomic DNA was removed with DNaseI (Promega, USA) by the on-column method, described in the protocol. cDNA of all samples was synthesized from 500 ng of total RNA, using iScript cDNA Synthesis Kit (Bio-Rad, USA). Oligo-dT supplied with the kit was used as the primer for the first-strand synthesis. CLeAPS-F (5′-ATGGCGGCGACGAGCTTC-3′) and CLeAPS-R (5′-TCATATGATGGTTCCGCTAGGG-3′) primers were used for amplification of *LeAPS* cDNA, while CLeAPL1-F (5′-ATGGCGCTGCGGATTGAG-3′) and CLeAPL1-R (5′-TCAGATGACAAGGCCATCCTT-3′) primers were used for *LeAPL1* cDNA. The primer sequences and amplification cycles were according to the previous study^4^. Q5 High-Fidelity DNA polymerase (New England Biolabs, USA) was used for the amplification of full-length cDNA. The nucleotide sequences were compared with those of *L. punctata* strain 0202, using blastn. Multiple alignment analysis was performed between the deduced amino acid sequences of *LeAPS* and *LeAPS* and their homologs, using Clustal Omega (https://www.ebi.ac.uk/Tools/msa/clustalo/).

### Characterization of *LeAPS* and *LeAPL1* genomic fragments

Q5 High-Fidelity DNA polymerase (New England Biolabs, USA) was used for the amplification of *LeAPL1* and *LeAPS* genomic fragments. CLeAPS-F and LeAPS-R (5′-GCCCAATCCGAGCATTCT TAT-3′)^4^ primers were used for amplification of partial *LeAPS* genomic fragment, while CLeAPL1-F and CLeAPL1-R primers were for genomic *LeAPL1*. The amplified cycles were: 95 °C for 5 min; 40 cycles of 95 °C for 30 sec, 60 °C for 30 sec, and 72 °C for 90 sec; 72 °C for 5 min. The amplified fragments were cut from an agarose gel and purified using FavorPrep GEL/PCR Purification Kit (Favorgen, Taiwan). Subsequently, the fragments were ligated with the pGEM-T vector (Promega, USA) and cloned in *Escherichia coli* strain DH5-α. Recombinant plasmids were extracted, using FavorPrep Plasmid DNA Extraction Mini Kit (Favorgen, Taiwan). Universal primers M13F (−20) and M13R (−40) were used for initial DNA sequencing. Additional primers were designed based on the sequences and used to complete the sequences. The genomic sequences of *LeAPS* (accession number: MK878513) and *LeAPL1* (MK878512) were deposited in GenBank database.

### Gene expression analysis

Total RNA was extracted from 100 mg of strain 5632 fronds, collected at each time point. The extraction and cDNA synthesis followed the methods mentioned above. Quantitative reverse transcription PCR was done using SensiFast Real-Time PCR kit (Bioline, USA), on CFX96 Touch Real-Time PCR Detection System (Bio-Rad, USA). New primers for quantification of *LeAPS* and *LeAPL1* expression were designed. APSQ-F1 (5′-TCCCAGATTTCAGCTTCTATGATCGG-3′) and APSQ-R1 (5′ TGAATCTTGCAGTTCTTAATCACGC-3′) primers were for *LeAPS*, while APL1Q-F1 (5′-AGAACTCGAAGATCAGGAACTGC-3′) and APL1Q-R1 (5′-TCTTTCGGCTTCTTGGATTCCCTC-3′) primers were for *LeAPL1*. Previously described primers were used to quantify expression of *LeAPL2* (LeAPL2-F/LeAPL2-R) and *LeAPL3* (LeAPL3-F/LeAPL3-R)^4^. Gene expression levels were determined according to a previously described method^28^. Briefly, five μl of cDNA of all samples were combined. A standard dilution series of 1x, 10^−1^X, 10^−2^X and 10^−3^X concentrations were made. The quantitation cycle (C_q_) values of the standards and all samples were determined. Standard curves of standard cDNA concentrations and C_q_ values were generated. Gene expression levels of all samples were determined by normalizing the sample C_q_ values against the standard curves. The experiment was performed in triplicate. Two-tailed student’s *t*-test was used to determine statistically significant differences (*P* < 0.05) between expression levels of each AGPase subunit genes in MS and Hoagland’s media on each day. Correlation coefficients between the averages of starch content and expression levels were determined for each gene.
